# MUC1 and glycan probing of CA19-9 captured biomarkers from cyst fluids and serum provides enhanced recognition of ovarian cancer

**DOI:** 10.1038/s41598-025-86735-z

**Published:** 2025-01-25

**Authors:** Shamima Afrin Ruma, Rufus Vinod, Shruti Jain, Kaisa Huhtinen, Johanna Hynninen, Janne Leivo, Kim Pettersson, Karin Sundfeldt, Kamlesh Gidwani

**Affiliations:** 1 Department of Life Technologies, Division of Biotechnology, University of Turku, Medisiina D, 5th floor, Kiinamyllynkatu 10, 20520 Turku, Finland; 2 Institute of Biomedicine and FICAN West Cancer Centre, University of Turku and Turku University Hospital, Turku, Finland; 3 InFLAMES Research Flagship, University of Turku, 20014 Turku, Finland; 4 Department of Obstetrics and Gynecology, Institute of Clinical Sciences, Sahlgrenska Center for Cancer Research, University of Gothenburg, Gothenberg, Sweden

**Keywords:** CA19-9, MUC1, MUC16, Europium nanoparticles, Epithelial ovarian cancer, CYST fluids, Biotechnology, Cancer, Biomarkers, Oncology

## Abstract

Glycosylation changes of circulating proteins carrying the CA19-9 antigen may offer new targets for detection methods to be explored for the diagnosis of epithelial ovarian cancer (EOC). Search for assay designs for targets initially captured by a CA19-9 antigen reactive antibody from human body fluids by probing with fluorescent nanoparticles coated with lectins or antibodies to known EOC associated proteins. CA19-9 antigens were immobilized from ascites fluids, ovarian cyst fluids or serum samples using monoclonal antibody C192 followed by probing of carrier proteins using anti-MUC16, anti-MUC1 and, anti STn antibodies and seven lectins, all separately coated on nanoparticles. Compared to reference CA19-9 and CA125 immunoassays, nanoparticle aided detection using MUC16, Ma695 and STn antibodies and lectin WGA provided, both separately and combined, improved discrimination of EOC and borderline cancers from benign samples when applied to 60 cyst fluid specimens. When applied to a panel of 44 serum samples (EOC N = 24, healthy and benign samples N = 20) two assays, CA19-9^Ma695^ and CA19-9^MUC1^, stood out with equally superior separations (p-values < 10^–8^) of the two groups compared to conventional CA19-9 immunoassay (p-value 0.03).Eu^+3^ -NP based CA19-9^MUC16^, CA19-9^Ma695^, CA19-9^STn^ and CA19-9^WGA^ show promise for improved EOC detection when applied to ascites & cyst fluids. When applied to circulation-derived samples, the two MUC1 based assays, CA19-9^Ma695^ and CA19-9^Ma552^ outperformed other assay constructs. Our results call for further validation in larger EOC cohorts preferentially with early stage ovarian cancers and all major histotypes against commonly occurring benign conditions.

## Introduction

Epithelial ovarian cancer (EOC) is the deadliest form of gynecological malignancies because of late and vague symptoms of the early disease states^1^. Transvaginal ultrasonography (TVU) and quantitative measurement of CA125 enable detection of mostly advanced EOC^2^. CA125, a large transmembrane mucin (MUC16), heavily glycosylated with both N- and O-linked glycans, is approved for monitoring EOC progression and therapeutic response^3,4^ but has a low diagnostic specificity because elevated levels of CA125 are frequently increased in several benign gynecological conditions and in non-gynecologic cancers, like pancreatic and lung cancer^5^. Early detection remains challenging although several biomarkers like HE4, cancer antigens 19–9 and 15–3 (CA19-9, CA15.3) have been tested in combination with CA125^6,7^ . Hence, new approaches are needed for early detection of EOC.

EOC frequently secrete glycoproteins and glycolipids carrying glycan structures such as sialylated Tn antigen (STn) and sialyl-Lewis A (sLeA)^8–10^. The sLeA glycan, which is usually attached to O-glycans on the surface of cells forms the basis for the CA19-9 test, named after the monoclonal antibody first developed against the sLeA antigen. Commercial CA19-9 tests are heterologous immunoassays using the same antibody for capture and detection. CA19-9 is the most used and best-validated serum tumor marker for pancreatic cancer in symptomatic patients, and strictly correlates with the clinical response after pancreatectomy^11^. The most common protein carriers of CA19-9 antigen in pancreatic cancer are MUC16, MUC1 and MUC5AC. In several benign and malignant conditions, these and other proteins may shift and affect the diagnosis tests performance^12–14^. For example, combination of MUC5AC or MUC16 with CA19-9 antigen improved diagnostic performance in discriminating pancreatic cancer from benign conditions over the CA19-9 assay alone^15^.

In search for new biomarkers, serum remains the most common source, but proteomic profiling of other biological fluids have also been evaluated^16–19^. Ascites fluid is a source rich in various proteins, lipids and exosomes secreted directly by ovarian cancer cells, and hence can be of help in identifying new diagnostic targets for early stage EOC^16,20–22^. Ovarian fluid filled cysts or sacs are commonly formed both normally as well as in benign and malignant conditions. Very early pathological changes in ovarian cysts may disclose proteomic patterns involved in tumorigenesis even before secretion into blood. Therefore, cyst fluids can be used as direct targets to find or evaluate new biomarkers especially for identifying early stage ovarian cancer^22^. Representing more invasive measures, neither ascites nor cyst fluids qualify as routine samples for identification of EOC, and any marker candidates present in these need to be validated in samples from the blood circulation.

Typical cancer-associated changes in glycosylation include truncated *O-*glycans, altered sialylation, fucosylation (mainly core fucosylation), including overexpression of sialyl Lewis x (sLeX) and sialyl Lewis a (sLeA), and are associated with tumor progression, metastatic spread, and poor prognosis^20,21^. Various glycan binding proteins (like lectins) and antibodies can be used as tools to detect altered glycosylation in cancer^23^. We previously reported that human recombinant lectin MGL and anti-STn antibody coated on fluorescent europium (III)-chelate dyed nanoparticles (Eu^3+-^NPs) detects EOC specific glycovariant (GV) of CA125 (MUC16^STn^ , MUC16^MGL^) and CA15-3. In the clinical evaluation, the resulting optimized assay showed good discrimination between serum samples from EOC and patients and patients with various benign conditions.^24–28^. However, in serum, benefits are found particularly in postmenopausal women, yet some early stage cases still remain undetectable^28^. Moreover, the claimed lack of CA125 or STn expression in individual patients in several cancers, calls for complimentary alternative biomarkers other than CA125.

In search for additional EOC biomarkers, we used a highly sensitive Eu^+3^-nanoparticle test approach to screen for carrier proteins and lectin identified glycostructures co-occurring with the CA19-9 antigen. Using the highly concentrated ascites and cyst fluids for initial discovery of promising biomarker combinations we subsequently proceeded to a limited scale verification of their diagnostic performance using serum samples from EOC patients and healthy and benign controls against conventional CA125 and CA19-9 references.

## Results

### Screening of EOC specific carrier proteins and glycans on CA19-9 in ascites

The biotinylated monoclonal antibody-C192 was used to immobilize CA19-9 carrying targets from ascites of EOC (n = 5) and LC (n = 2) subjects. The 5 EOC ascites were collected from HGSOC, pretreatment patient samples. The immobilized CA19-9 was probed with a small panel of different glycan binding lectins such as WGA, UEA, MBL and DC-sign or antibodies such as STn, MUC16, and Ma695 coated on Eu^+3^-NPs. Of the eight combinations tested with this small panel, seven appear preferentially recognizing the EOC specimens with the Ov185 (MUC16) and Ma695 (MUC1) giving the best separations. The DC-Sign NPs were excluded from further testing. A column graph is generated to represent the specific signals ratio of the CA19-9 based glycovariant and carrier protein assays with ascites fluid samples of malignant and non-malignant (Data has been shown as supplementary).

### Evaluation of EOC specific CA19-9 candidates in ovarian cyst fluid

**Fig. 1:**
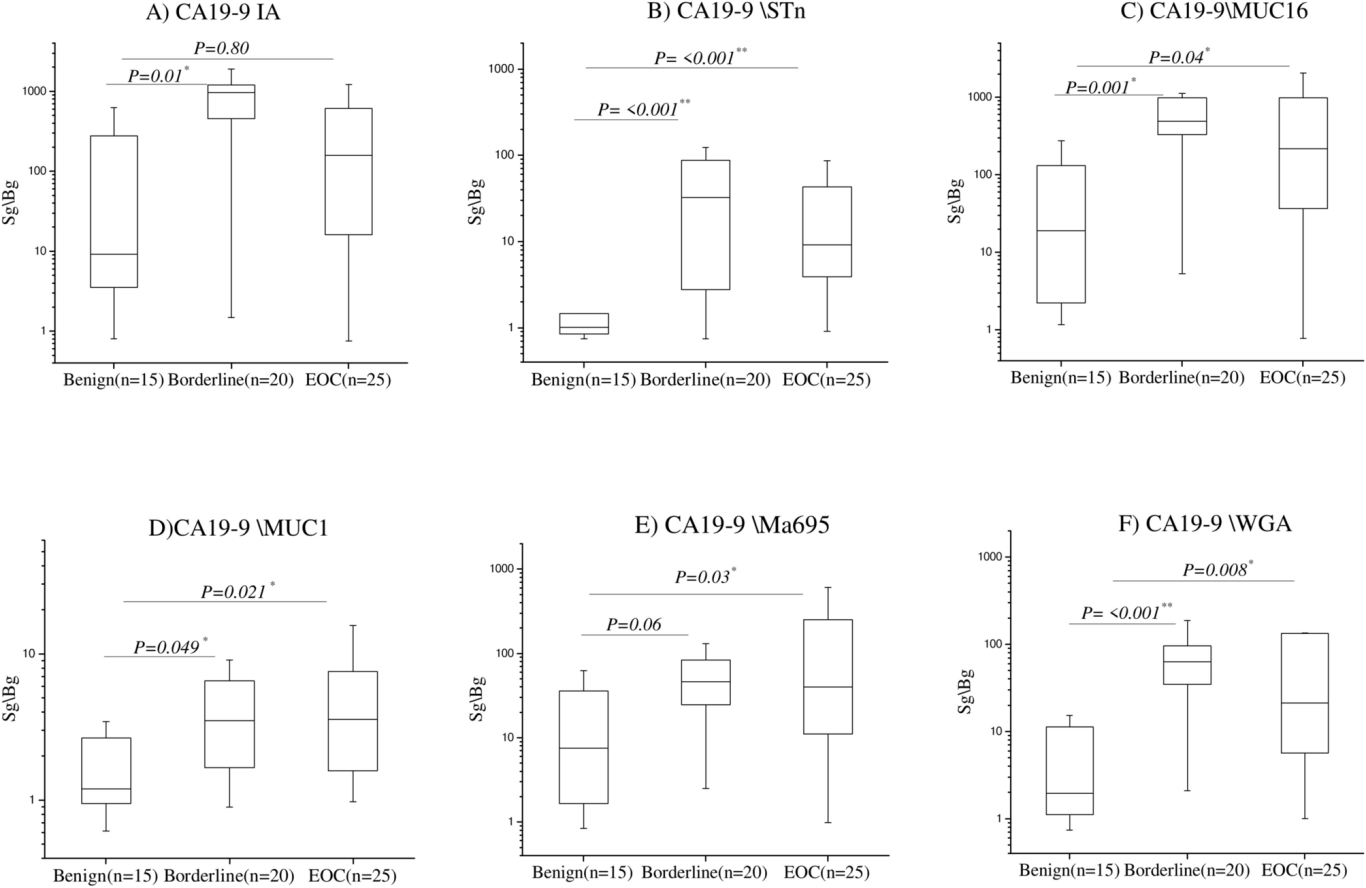
CA19-9 glycovarients CA19-9^STn^, CA19-9^MUC16^, CA19-9^Ma695^, CA19-9^WGA^ (**B**, **C**, **D** & **F**) discriminates significantly borderline (n = 20), type1 and type 2 EOC (n = 25) patients from benign control (n = 15) of ovarian cyst fluid. * = significant, ** = highly significant. Bonferroni correction for multiple test has been used for adjusted P values.

The combinations selected from the ascites test were further evaluated with a cohort of 60 ovarian cyst fluid samples. Results from CA19-9^STn^, CA19-9^MU16C^, CA19-9^MU1C^, CA19-9^Ma695^, CA19-9^WGA^ and the CA19-9^IA^ reference are shown in (Fig. 1).

Whereas the reference CA19-9 IA does not separate EOC from the benign group due to the large spread of the latter, all the new glycovariants do. Also, four CA19-9 glycovariants (B, C, and D&F) show significant discriminations of the benign and borderline groups, albeit with significant spread in either the benign or the borderline groups.

**Fig. 2:**
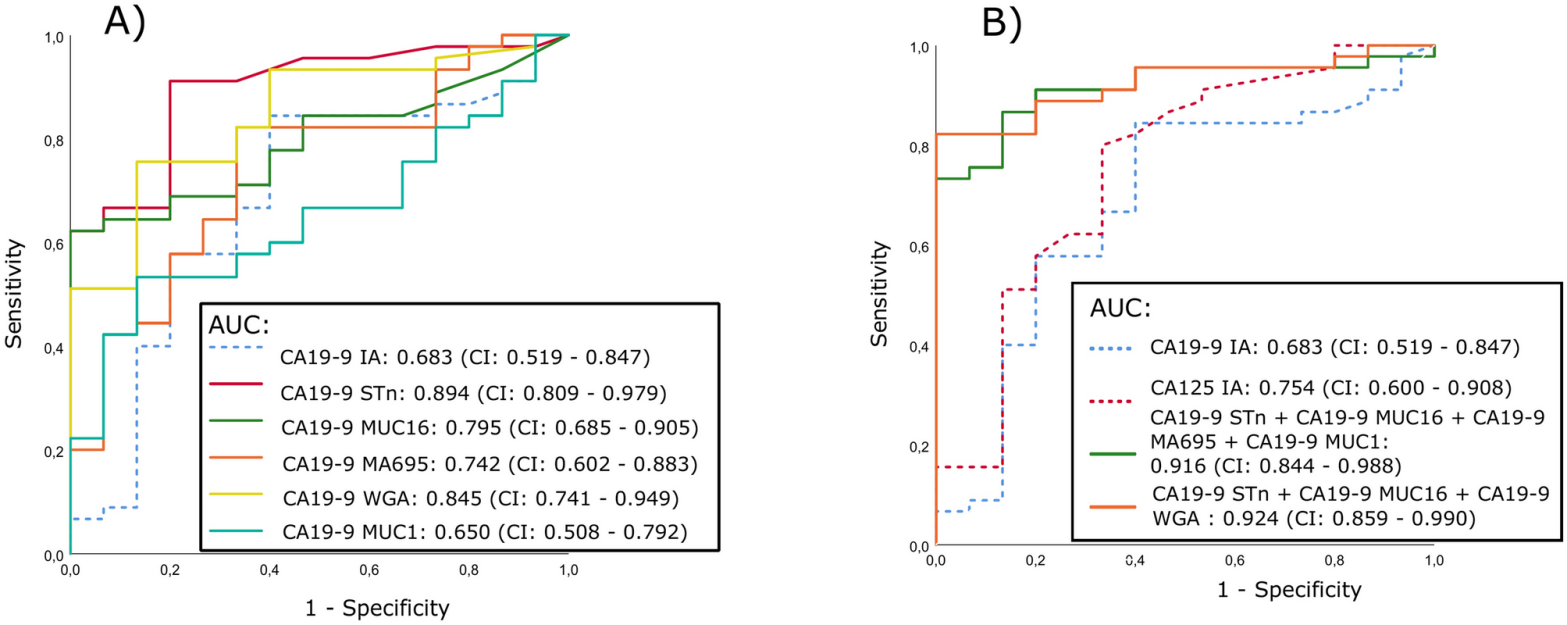
Receiver Operation Characteristics of conventional and glycovariants assays. The S\B ratios of the conventional assays (CA19-9^IA^) and the glycovariant assays were used for classifying benign (n = 15) and epithelial ovarian cancer patients(n = 45) of cyst fluid samples (A) ROC displaying the area under curve (AUC) of individual CA19-9^IA^ (dotted line) & their glycovariant CA19-9^STn^ CA19-9^WGA^ CA19-9^Ma695^ CA19-9^MUC1^and MUC16 detecting carrier protein CA19-9^MUC16^ assay (Solid line). (B) ROC plot showing CA19-9 ^IA^ & CA125^EIA^ (dotted line) & combination of CA19-9 STn, CA19-9^MUC16^ & CA19-9 ^WGA^ and combination of CA19-9^STn^, CA19-9^MUC16^, CA19-9^MUC1^ & CA19-9^Ma695^ of glycovariant (green solid line). Data information: AUC with 95% CI is stated in figure diagram.

In order to evaluate and illustrate the diagnostic performance especially at high specificities we chose to perform ROC analysis of the combined EOC and borderline groups (n = 45) against the benign cases (n = 15) for the six individual assays (Fig. 2A) and for combinations of three or four assays (Fig. 2B).

#### ROC analysis of CA19-9^MUC16^, CA19-9^Ma695^, CA19-9^MUC1^, CA19-9^STn^, CA19-9^WGA^, ***a***ssays in ovarian cyst fluid

In ROC analysis, the AUC of CA19-9^IA^ was 0.683, which increased to 0.894 with CA19-9^STn^ GV, followed by CA19-9^WGA^, CA19-9^MUC16^, and CA19-9^Ma695^. At 95% specificity (SP), the sensitivity (SN) of CA19-9^IA^ increased from 8.9% to 42,2% for CA19-9^MUC1^.The AUC of CA19-9 glycovarients, CA19.9-^MUC16^, -^Ma695^, -^WGA^ -are higher (0.795, 0.742 & 0.845 respectively) than CÁ19-9IA (Fig. 2A). Additionally, the combination of CA19-9^STn^ + CA19-9^MUC16^ + CA19-9^WGA^ and combination of CA19-9^STn^ + CA19-9^MUC16^ + CA19-9^Ma695^ + CA19-9^MUC1^suggest great additional value with AUC 0.924 & 0.916 respectively. At 95% specificity all the combination increased their sensitivity to 82.2% & 75.6% than CA19-9 IA (Fig. 2B).

### Serum CA19-9^EIA^, CA19-9^Ma695^, CA19-9^MUC1^ assays to detect patients with EOC and controls

**Fig. 3:**
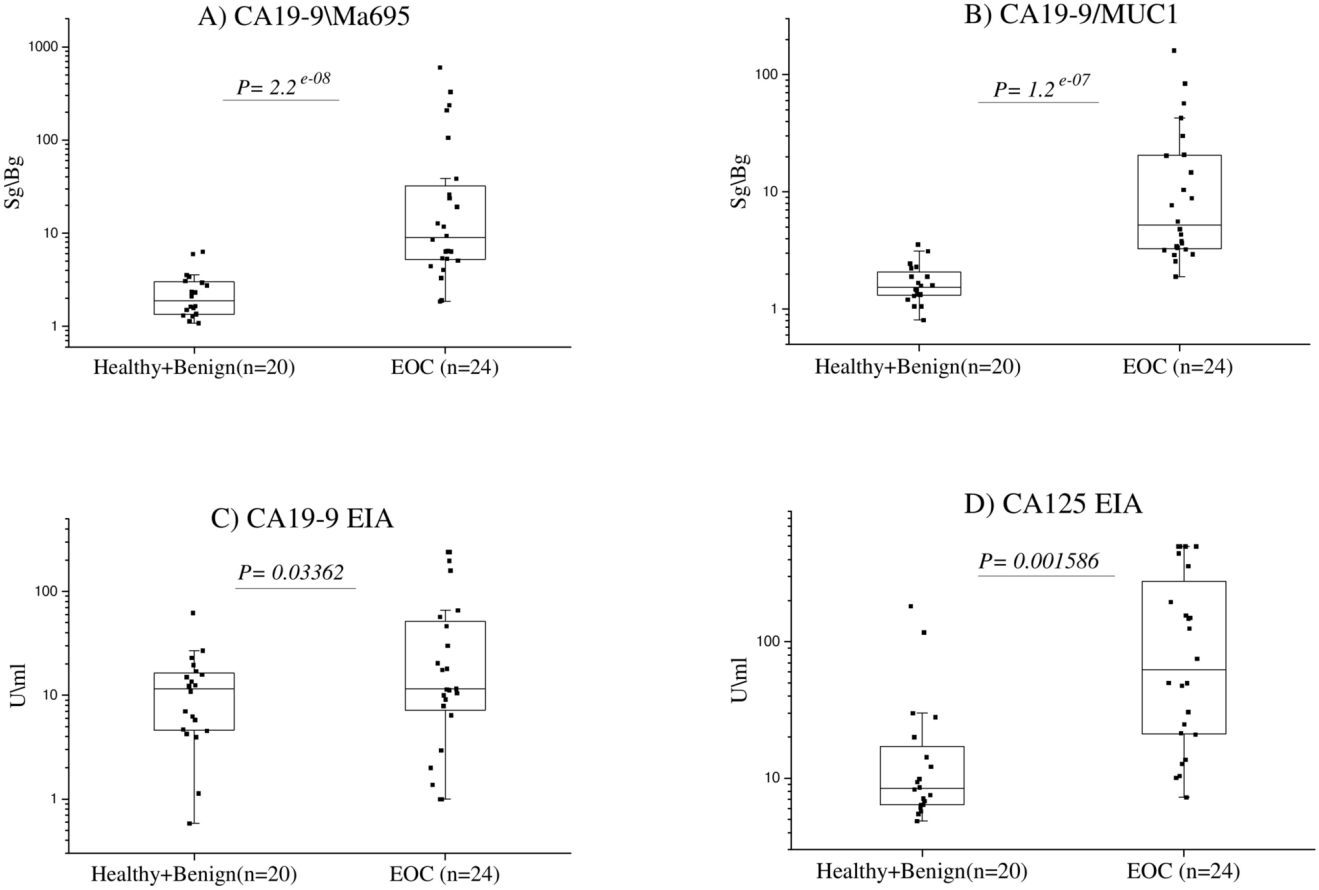
Discrimination of EOC (n = 24) patients from healthy (n = 15) and benign (n = 5) controls of CA19-9 glycovarients CA19-9 ^Ma695^ and MUC1 detecting carrier protein CA19-9 ^Ma552^ assays in patient serum samples.

**Fig. 4:**
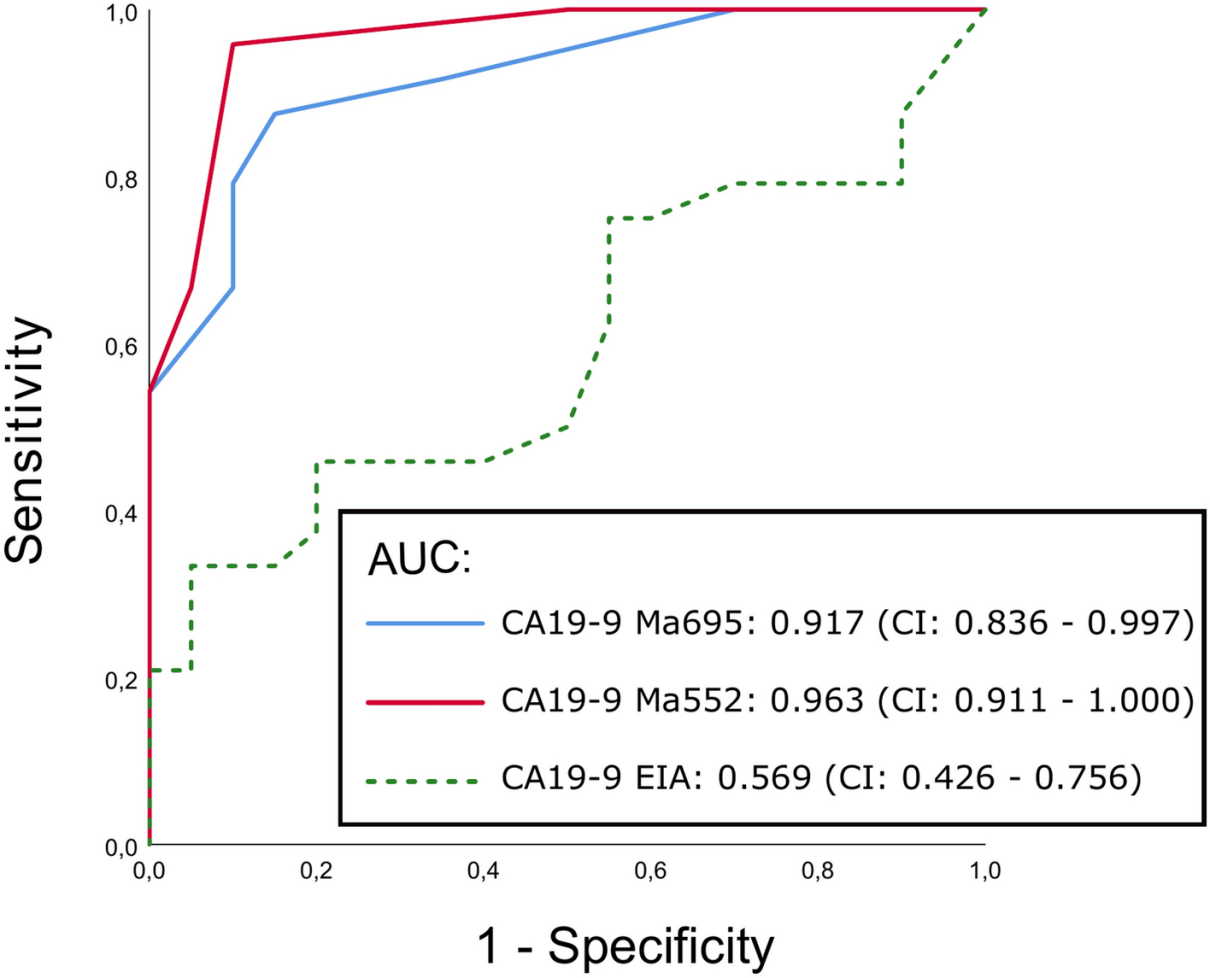
ROC plot of serum CA19-9 glycovarient assays for the detection of EOC.

In a final step, we proceeded to evaluate the novel CA19-9 markers with a panel of serum samples from 24 EOC patients, 15 healthy and 5 benign controls. The two assays CA19-9^Ma695^ & CA19-9^Ma552^ employing for detection two different MUC1 antibodies coated to Eu^+3^ nanoparticles outperformed the other assay combinations as shown in Fig. 3 in comparison with the CA19-9 and CA125 reference assays. In the ROC curve analysis, at 90% specificity both the CA19-9 based glycovarient assays CA19-9^Ma695^ and CA19-9^MUC1^ has shown superior sensitivity (79.2% and 95.8% respectively) over the conventional CA19-9^EIA^ (33.3%). Additional ROC analysis illustrates the diagnostic performances of CA19-9 glycovarients in serum of EOC samples (Fig. 4).

## Discussion

Circulating cancer specific glycoprotein biomarkers have great potential for early detection of cancer but that potential is yet to be realized. We have previously utilized lectin or anti-glycan antibody conjugated europium-nanoparticles (Eu^+3^-NP) successfully to explore the glycosylation of conventional tumor biomarkers; CA125, CA15-3, PSA as well as urinary extracellular vesicles for ovarian, breast, prostate and bladder cancer respectively^23,25,29^. The sialyl Lewis a glycan forms the basis for the CA19-9 test, which is the most used serum tumor marker for monitoring of pancreatic cancer. However, few studies have addressed their utility in epithelial ovarian cancer (EOC) in combination with CA125. CA19-9 was recently reported to add value to CA125 for the detection of mucinous-type ovarian malignancies^30^. In this work, we sought to find CA19-9 antigen expressing markers in EOC patients through detection of either the carrier protein or an associated glycan structure. We found that CA19-9 expressing markers when immobilized from EOC ascites secretions could be detected with MUC16 mAb (CA19-9^MUC16^), Ma695 mAb (CA19-9^Ma695^), STn mAb (CA19-9^STn^) and mannose, terminal GlcNAc, fucose -binding lectins MBL (CA19-9^MBL^), WGA (CA19-9^WGA^) and UEA (CA19-9^UEA^) respectively.

Upon further evaluation using 60 ovarian cyst fluids, five CA19-9 based marker candidates CA19-9^STn^, CA19-9^MUC16^, CA19-9^MUC1^, CA19-9^Ma695^ and CA19-9^WGA^ were identified providing improvement over conventional CA19-9 IA. This study included 45 surgery requiring EOC or borderline tumors and 15 healthy controls. Especially the combination of the new assays in ROC analyses indicated substantial improvement in AUC compared to the conventional CA19-9 immunoassay especially in the high specificity area.

The rationale for selecting ascites and cystic fluids for the discovery part of this study is three- fold. First, these fluids are thought to more closely reflect the marker changes derived from the tumor. Secondly, the biomarker concentrations are higher than those in the circulation allowing more extensive and repetitive testing from the same specimens. Thirdly, these fluids lack potentially cross-reacting and interfering components from the highly heterogenous circulating specimens. On the other hand, neither ascites nor cyst fluids are convenient, suitable or realistic samples for diagnostic routines requiring easily available non-invasive samples such as serum, plasma or urine.

Consequently, after the discovery and evaluation work especially using cyst fluids we further evaluated CA19-9 associated assays in serum samples of 24 EOC and 20 healthy and benign samples as controls. The Ma695 and Ma552 mAb detect sialylated carbohydrate and protein epitope of MUC1 respectively. Both assays correlate closely with each other in this panel suggesting that Ma695 recognizes its sialylated carbohydrate glycan exclusively on MUC1.

Thus, the several promising glycovariant candidates initially identified from the ascites and cyst fluid panels, could not convincingly be verified when switching to the final, albeit limited serum panel. The likely explanation of this is that both the sialylated CA19-9 antigen as derived from capture with the C192 Mab as well as the glycan motifs identified by the lectins or STn antibody on the EU^+3^ nanoparticles can be found on a wide variety of proteins in the circulation. Previous use of CA19-9 using two-site homologous immunoassays for EOC diagnosis has showed limited contribution over traditional CA125^31^. Our study strongly shows that combining the capture of CA19-9 with the two MUC1 antibodies provides a decisive improvement in separating the EOC samples from the controls. In this limited cohort, we did not have information about individuals genotypically negative for the Lewis a antigen (about 5–10 percent of Caucasians population). This limitation should be addressed in future more extensive studies.

Ricardo et al.reported that MUC16 and MUC1 are major carrier proteins of SLeA in EOC tissue lysates using proximity ligation assays^32^. Our study shows the same both in using ascites or cyst fluids but more importantly using serum samples with a use of a simple and robust Eu^+3^NP based immunoassay. This study was primarily designed to identify novel EOC biomarker candidates using the CA19-9 antigen capture approach to seek further improvement of detection of early EOC stages or histotypes that are missed with the traditional CA125 or its STn glycovariants either due to representing non expression cancers) or testing negative due to blocking by autoantibodies^33^. From the limited serum sample evaluation we noticed several patient samples testing negative with our previously reported CA125^STn^ while being easily detected with the CA19-9^MUC1^.

The limited serum sample cohort is a shortage of this study and clearly calls for a substantially larger cohort of EOC samples representing both different cancer stages and histotypes as well as including samples from normally confounding benign gynecological conditions. Such a cohort has been identified for extending this study and will include analysis both by reference CA125 and HE4 as well as our previously published STn and MGL glycovariants of MUC16 and MUC1.

As both MUC1 and CA19-9 antigen are expressed in many cancer types^11,34–36^ it will also be necessary to evaluate the suggested CA19-9^MUC1^ assay with such cancers and relevant benign conditions.

Our results suggest that the novel CA19-9 based assays CA19-9^MUC1^ and CA19-9^Ma695^ as initially identified from ascites and cyst fluids show a decisive improvement over the conventional CA19-9 EIA when applied to the detection of EOC. Because the serum samples available to this proof-of-concept bio-discovery report are limited, these novel CA19.9-MUC1 assays need to be evaluated on substantially larger EOC patient cohorts and relevant benign conditions.

## Materials & methods

### Patient characteristics and sampling

This study included the ascites fluid samples (n = 7) from five preoperative EOC and two liver cirrhosis patients as control.

Further this study included a cohort of 60 preoperative ovarian cyst fluid samples. This cyst fluid cohort was previously described by Wang et al., 2016^37^. After initial screening, the shortlisted candidates were then tested on the whole cohort. Non-neoplastic cysts (n = 7) and benign tumors (n = 8) were classified as surgery not required group, whereas cysts associated with the borderline tumors (n = 20) and invasive cancers (malignant n = 25) need to be surgically removed. Finally, serum samples of 24 invasive epithelial ovarian cancer and 20 benign (n = 5) and healthy (n = 15) controls were analyzed.

### Reagents

The anti-CA19-9, -MUC16, -MUC1, -STn and Ma695 mAb were kindly provided by Fujirebio Diagnostics (Gothenburg, Sweden). Detail of antibodies are described in Table 1.The yellow streptavidin coated 96 well micro titer plates, wash buffer and the assay buffer were obtained from Kaivogen Oy (Turku, Finland). Europium (III)-chelate-doped Fluoro-Max™ polystyrene nanoparticles (95 nm in diameter) (Eu^+3^-NP) were acquired from Seradyn Inc. (Indianapolis, IN, USA). A panel of different glycan binding plant lectins were purchased from Vector laboratories (Burlingame, CA, USA). Hidex Sense from Hidex Oy (Turku, Finland) was used for the time resolved fluorescence (TRF) measurement.

**Table 1:** List of antibodies\lectins used for the detection of CA19-9 antigen motif in EOC associated fluids.

Antibody/lectin	Full name	Recognizes Protein or glycan epitope	Vendors
C192	Anti-CA19.9 antigen monoclonal antibody	Sialyl Lewis A glycans	Fujirebio
Ov185	Anti-MUCIN 16 antigen monoclonal antibody	Protein epitope of MUC16	Fujirebio
Ma552	Anti-MUCIN 1 antigen monoclonal antibody	Protein epitope of MUC1	Fujirebio
Ma695 mAb	Anti-sialylated glycans monoclonal antibody	Sialylated carbohydrate	Fujirebio
STn1242 mAb	Anti-STn antigen monoclonal antibody	Sialylated Tn Antigen	Fujirebio
MBL	Mannose binding lectin		RnD System
WGA	Wheat germ agglutinin	Terminal GlcNAc or chitobiose	Vectorlabs
UEA	Ulex europaeus agglutinin	Fucoseα1-2galactose	Vectorlabs
DC SIGN	Dendritic Cell-Specific Intercellular adhesion molecule-3-Grabbing Non-integrin	Nonsialylated Lewis antigens and high mannose-type structures	RnD System

### Preparation of biotinylated antibodies

With 40 molar excess of biotin isothiocyanate, anti-CA19-9 antibodies were biotinylated for 4 h at room temperature (RT) as described previously^23^. Biotinylated antibodies were purified using NAP™-5 and NAP™-10 gel-filtration columns (GE Healthcare, Schenectady, NY, USA) with the use of 50 mmol/L Tris–HCl (pH 7.75), containing 150 mmol/L NaCl and 0.5 g/L NaN_3._ The antibodies labelled with biotin were stored in 1 g/L BSA at 4 °C.

### Preparation of Eu^+3^-NP coated antibodies/lectins

The application of Eu^+3^-NP as bio-conjugated reporter has been described previously^38^. The amino groups of monoclonal antibodies (mAb) and glycan binding proteins (lectins) were covalently attached to the activated carboxyl group of the Eu^+3^-NPs. Eu^+3^-NPs conjugated antibodies\lectins were stored using the buffer 10 mM Tris–HCl, pH 7.8, supplemented with 0.1% BSA(Bioreba) and 0.01% sodium azide at + 4 °C. Before using, the particles were thoroughly vortexed every time in order to disperse any big aggregates.

### In-house time resolved fluorescence (TRF) assay

Procedures for the measurement of the cyst, ascites and serums were done similarly as follows. The biotinylated anti-C192 monoclonal antibody was immobilized 40 ng/25 µl/well on streptavidin coated microtiter plate in assay buffer for 1 h at RT without shaking. After washing the plate twice with wash buffer, the cysts or ascites or serums were added 25 µl/well in triplicates with 1:10 dilution in assay buffer and incubated for 1 h at RT with slow shaking. After incubation the wells were again washed twice with wash buffer and the lectin or antibody conjugated Eu^+3^-NPs were added 1e7/25 µl/well and incubated for 1 h at room temperature (RT) with slow shaking. The tracer wash was done 6 times and the TRF measurement was taken in Hidex Sense (λ_ex_ = 340 nm and λ_em_ = 615 nm).

### Statistical analysis

The column analysis of ascites fluid screening was done using Origin 2016. RStudio. The box plot analysis for the cyst fluids was performed with Origin 2016. R Studio was used to calculate the P-values using the two-sample t-test & Wilcox test, where P-value below 0.05 was considered significant (R Core team, 2015) software with ggplot2 (Wickham & Chang, 2015) R packages. Receiver operating characteristics (ROC) were determined and compared, and the areas under the curve (AUC) values were calculated using SPSS version 23 statistical software package (SPSS Inc., Chicago, IL, USA).

## Supplementary Information

Supplementary Information.

## Data Availability

The data that support the findings of this study are available from the corresponding author upon reasonable request.

## Abbreviations

AUC: Area under the curve
BSA: Bovine serum albumin
CA125: Cancer antigen 125
CA19-9: Cancer antigen 19–9
CF: Cyst fluid
CI: Confidence interval
EOC: Epithelial ovarian cancer
Eu-NP: Europium (III)-chelate-dyed nanoparticles
GV: Glycovariant
IA: Immunoassays
LC: Liver cirrhosis
mAb: Monoclonal antibody
n: Number
NP: Nanoparticles
ROC: Receiver Operating Characteristic
RT: Room temperature
S/B: Signal/background
SN: Sensitivity
SP: Specificity
STn: Sialyl-Tn antigen
TRF: Time-resolved fluorescence
HGSOC: High grade serous ovarian cancer
