# Supplementary material for: MUC1 and glycan probing of CA19-9 captured biomarkers from cyst fluids and serum provides enhanced recognition of ovarian cancer
Source: Sci Rep. 2025 Jan 25;15:3171. doi: 10.1038/s41598-025-86735-z (PMC11762729; doi:10.1038/s41598-025-86735-z)
Supplement: Supplementary file 1 — Supplementary Information. [file 41598_2025_86735_MOESM1_ESM.pdf]

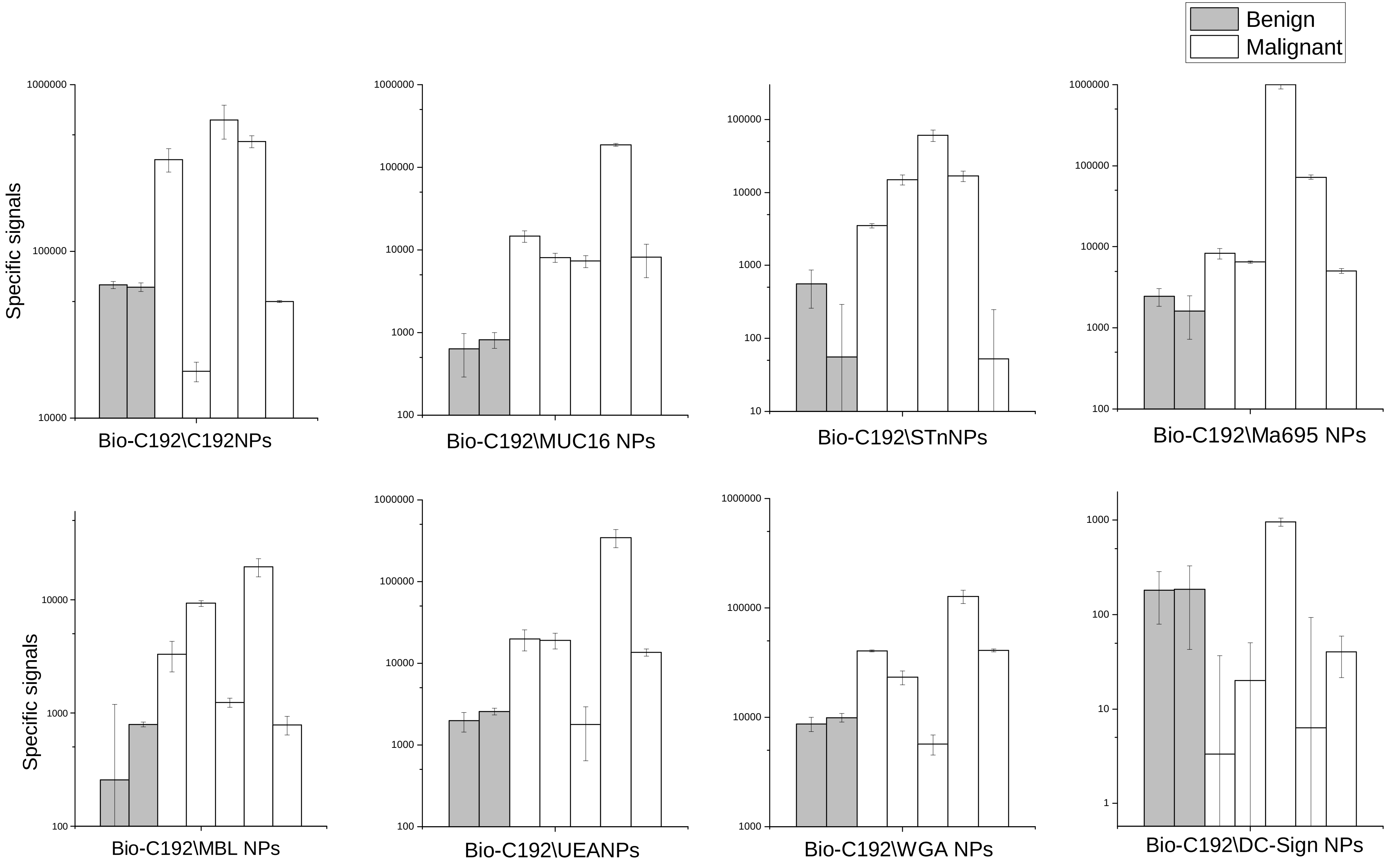

Supplementary figure 1: Characterization of CA19-9 to detect carrier proteins and associated glycans. The column graph represents the specific signals ratio of the CA19-9 based glycovariant and carrier protein assays with ascites fluid samples of malignant (n=5) and non-malignant, liver cirrhosis (n=2).
